# Correlation between goniometric measurements of range of motion and radiographic scores in osteoarthritis knee: An observational study among females

**DOI:** 10.1097/MD.0000000000029995

**Published:** 2022-08-12

**Authors:** Md. Rashid Al-Mahmood, Md. Taslim Uddin, Mohammad Tariqul Islam, Shamim Md Fuad, Tanvir Rahman Shah

**Affiliations:** a Department of Physical Medicine and Rehabilitation, Bangabandhu Sheikh Mujib Medical University (BSMMU), Shahbag, Dhaka, Bangladesh; b Northern International Medical College, Dhaka, Bangladesh; c Department of Physical Medicine and Rehabilitation, Asgor Ali Hospital, Dhaka, Bangladesh.

**Keywords:** female OA knee, goniometric measurements, osteoarthritis knee, radiographic score, range of motion of OA knee

## Abstract

Osteoarthritis (OA) is a chronic degenerative joint disease. Different radiological changes are found according to grades. Range of motions (ROMs) of knee decreases with severity of OA. Women are more sufferer than men in OA knee. Objective was to correlate goniometric ROM with Kellgren-Lawrence (KL) radiographic score of female osteoarthritic knee. The study was a cross-sectional study conducted in Department of Physical Medicine and Rehabilitation, BSMMU, Dhaka, from February 2020 to March 2021. According to ACR (American College of Rheumatology) criteria, total 66 patients with primary OA knee were selected and examined in this study. Maximal flexion, extension, and rotation movements were measured by a universal goniometer. X-ray of standing both (A/P and lateral) view and skyline view of knee joint were taken and assessed with KL radiographic scores for medial, lateral, and patellofemoral compartments. Correlations between ROMs and KL scores were analyzed by Pearson correlation test. Among the 66 patients, mean age was 53.59 ± 7.19 years and mean body mass index was 26.62 ± 3.35. Majority (84.8%) of the patients were housewives. Mean maximum flexion was 126.71 ± 4.88°, maximum extension was –3.98 ± 1.74°, and internal and external rotations were 6.38 ± 1.29 and 8.48 ± 1.55°, respectively. More than half of patients had medial compartment KL score 3 or more while KL score 2 was found in 47% and 62.1% patients, respectively, in lateral and patellofemoral compartments. Statistically significant negative correlations were found between range of motion and radiographic scores. Strong correlation was present between maximal flexion and medial compartment score (r = –0.821, *P* < .001), whereas moderate correlation with other compartments. Extension values were moderately correlated with patellofemoral scores (r = –0.560, *P* < .001) and weakly correlated with rest of radiographic scores. Internal and external rotation were more related with medial compartment (r= –0.469, *P* < .001) and lateral compartment scores (r = –0.481, *P* < .001), respectively, than other compartment scores. There were significant negative correlations between goniometric measurements of knee ROM and radiographic scores in osteoarthritis knee in female patients.

## 1. Introduction

Osteoarthritis (OA) is one of the most common cause of disability, and the knee joint is the most common site for lower extremity OA.^[1]^ Women are affected more frequently than men in osteoarthritis knee. As per WHO estimates, the prevalence of knee OA was 1770 and 2693 per 100,000 men and women in 2000, respectively.^[2]^ Prevalence of osteoarthritis (OA) knee is 7.5% in rural; 6.4% men; and 8.5% women, whereas 10.6% in urban affluent community; 6.3% men and 15.9% women in Bangladesh perspectives.^[3]^ In Bangladeshi urban population, the prevalence of OA knee is high with increased body mass index (BMI). In rural community, cultivation and home making are associated with increased knee OA prevalence.^[4]^ The ICD-10-CM codes for OA knee is M17. Codes for bilateral primary OA knee is M17.0 and for unilateral primary OA knee is M17.1. For more specification, codes are M17.10-unilateral primary OA unspecified knee; M17.11-unilateral primary OA right knee; and M17.12-unilateral primary OA left knee.

Range of motion (ROM) testing help to assess the integrity of a joint, to monitor the efficacy of treatment regimens, and to determine the cause of an impairment. Limitations of motion affect ambulation, mobility, and ADL.^[5]^ Sufficient ROM of knee is important for ADL such as standing up from chair, walking, squatting, and stair climbing.^[6]^ With progression of OA, there is increased physical limitations, pain, and functional restrictions.^[7]^ Restricted flexion of knee is a strong risk factor for locomotor disability.^[8]^ Reduced knee ROM is also a predictor of both the incidence of OA and the progression of preexisting cartilage deficit.^[6–8]^ Although reduced knee motion is not included in the ACR criteria for the classification and reporting of OA of the knee, http://links.lww.com/MD/G991, it is part of 2 of the 10 European League Against Rheumatism (EULAR) recommendations for the diagnosis of knee OA.^[9]^

Different radiological classification system for OA is present. Among them Kellgren-Lawrence (KL) radiographic grading scale is a reliable method that is widely used in diagnosis and assessment of progression in knee OA.^[10]^ This score is from 0 to 4, where grade 0 indicates a definite absence of x-ray changes, grade 1 is doubtful, grade 2 indicates definite OA with minimal severity, whereas grade 3 is moderate and grade 4 is severe OA.^[11]^

Goniometric measurement of ROM is an important part of clinical examination in osteoarthritis knee and can give information about available knee joint motion, guide about pattern of management, and prognosis. Also, radiographic scores of knee joint compartments in patients with osteoarthritis knee give idea about compartment wise diagnosis and severity of disease. Very limited studies are available regarding correlating these 2 parameters and considering females as study population.^[6,9,10]^ Subjects of those previous studies also differ with female of Bangladesh regarding demographic and socioeconomic conditions. Women of Bangladesh mostly involve in home making, weight carrying, using low commode, cultivation (rural areas), work with poor posture or perform many ADL, which need excess and repetitive knee bending and twisting, which are greater risk to develop OA knee. This research will be helpful to provide evidence-based information about radiological grading of OA knee with variation of ROM. It may help to make step wise plan for specific management and rehabilitation, which will limit future disability, deformity, movement restriction, and deterioration of quality of life.

Hypothesis of the current study was “There is correlation between goniometric measurements of ROM and radiographic scores in osteoarthritis knee.”

Objective of the study was—to measure ROM and correlate with KL radiographic score of osteoarthritic knee.

## 2. Methodology

This was a descriptive cross-sectional study conducted in department of physical medicine and rehabilitation of a tertiary hospital in Dhaka from February 2020 to March 2021. Equation used for sample size calculation was N = [(Z_α_+Z_β_)/C]2 + 3. To obtain this, value of “r” was required, which was taken from “correlation coefficient” between maximum flexion and KLs score of medial tibiofemoral joint (=0.338).^[10]^ Total sample size was 66.

Sixty-six females, of 40 to 70 years age group with primary OA knee, fulfilling the ACR (American College of Rheumatology) criteria for knee OA, were selected purposively by the researcher. If bilateral OA knee present, the worse one was taken according to higher KL score and lower degree of active knee movements; if both knees were same osteoarthritic then randomly 1 was chosen. KL score was >1 at least in any compartment. Patients who received intra-articular corticosteroid, PRP, or viscosupplement to the target knee over previous 6 months; had history of previous knee surgery; severe medical comorbidities or moderate to severe knee pain (VAS > 6); had any inflammatory arthritis or trauma to knee; neuropathy or any neurological deficit of lower extremities, were excluded. A written informed consent was taken from individual patient with explanation of procedures. Proper screening and presence of female attendance were ensured.

### 2.1. Goniometric examination

A half circle long-arm metallic goniometer, ranging from 0 to 180°, with 1° interval marking was used. It had a central fulcrum, a stationary or fixed arm, and a pivoting or moving arm. Both arms were 30 cm long. The active ROM of knee joint was measured. Participants carried out the motion by using muscle strength to increase the angle. The examiner did not provide support or apply any kind of force for the completion of the joint motion. Measurements of knee flexion and extension were obtained with subjects lying supine on an examination table. Central fulcrum of goniometer was placed over lateral epicondyle of femur, stationary arm was aligned proximally with lateral midline of thigh along length of femur, using greater trochanter as reference, moving arm was aligned distally with lateral midline of leg along length of fibula, using lateral malleolus as reference. For well demarcation, cross marks were given over the reference points by a temporary marker (when needed). A towel roll or small pillow was placed under ankle. Normal extended knee was in the 0° position. A positive ROM score for extension is used for hyperextension. A negative ROM score for extension mean a patient was unable to reach the 0° position. Patient was asked to actively flex knee. At the end of knee flexion, examiner used 1 hand to hold stationary arm, while other hand was used to align the moving arm of goniometer with lateral midline of leg. Examiner kneeled or sat on a stool to see the measurements of goniometer on eye level.^[12]^ The degree of maximum flexion, extension, hyperextension (if present) was recorded. The summation of maximum flexion and maximum extension (extension + hyperextension) was described as the total excursion range. It resembled the available ROM in sagittal plane in knee. Internal and external rotation of the tibia were measured in a seated position, with the hip and knee flexed in 90°. Tibiofemoral and ankle joints were aligned in the same line, and the fulcrum of the goniometer was positioned just above the tibiofemoral joint. Stationary or fixed arm of the goniometer was placed along the long axis of the thigh, and the moving arm was placed on parallel to the long axis of the foot, which rotated internally or externally. The foot was kept on the floor to restrict eversion and inversion during rotations. Patient was asked to move leg actively in medial and lateral direction to measure internal and external rotation of tibia, respectively.^[10]^ All the knee ROMs were measured only by researcher.

### 2.2. Radiographic evaluation

Kellgren and Lawrence score was determined for medial tibiofemoral, lateral tibiofemoral, and patellofemoral compartments of the knee joint from X-ray knee standing anteroposterior, lateral, and skyline view. Weight bearing/standing anteroposterior and lateral view were used to see both tibiofemoral joints, while lateral view and Skyline view helped to see patellofemoral joint. Scoring was done from 0 to 4 for each compartment. The highest score obtained in the 3 compartments was used as the maximum KLs.^[10]^ X-ray of all patients were done from radiology department of same institution (BSMMU). Each radiograph was evaluated according to KL grading by a single expert radiologist who was blinded to patient’s identity or details.

### 2.3. Data analysis

The data collected from the respondents were entered into SPSS (statistical package for social science) layout. The statistical analysis was conducted using IBM SPSS version 26 statistical software (IBM corporation, Armonk, NY). The findings of the study were presented by frequency, percentage in tables and graphs. Means and standard deviations for continuous variables and frequency distributions for categorical variables were used to describe the characteristics of the total sample. Ranges were given as appropriate correlations between ROMs and KL scores were analyzed by Pearson correlation test. Strength of correlation coefficient were determined according to Hebel and McCarter.^[13]^ A *P* value <.05 was considered statistically significant.

### 2.4. Ethical implication

Ethical clearance was taken from the Institutional Review Board (IRB) of BSMMU (ID-BSMMU/2020/418, date of approval-21-12-2019). There was no physical, psychological, and social risk to the patients. Informed and understood written consent were taken from every patient before enrollment. Privacy, anonymity, and confidentiality of data information identifying any patient were maintained strictly. Each patient enjoyed every right to participate or refuse or even withdraw from the study at any point of time. The study conforms to code of ethics of the world medical association (Helsinki Declaration).

## 3. Result

The mean age of the patients was 53.59 ± 7.19 years and mean BMI was 26.62 ± 3.35 kg/m^2^ (Table 1). Table 2 represents the details of goniometric measurements of knee ROM of the respondents. Distribution of the patients by KL radiographic score was presented in Table 3. Higher scores of medial compartments comparing to other 2 compartments were found. Pearson Correlation coefficients between the knee ROM values and KL radiographic score were determined and represented on Table 4. Significant negative correlations were found between the parameters. Strong negative correlation was found between maximum flexion and medial tibiofemoral KL scores and maximum KL scores while moderate negative correlation with lateral tibiofemoral KL scores and patellofemoral KL scores were noticed. Moderate negative correlation was present in between extension and patellofemoral KL score and weak negative correlation between extension and rest of radiographic scores. There was strong to moderate negative correlation between total excursion and knee joint compartments. Weak negative correlation was found in both internal rotations and external rotations with all radiographic scores.

**Table 1:** Distribution of patients by age and BMI (n = 66).

	Mean ± SD	Range (min–max)
Age (in yrs)	53.59 ± 7.19	40–70
BMI (in kg/m^2^)	26.62 ± 3.35	(19.43–33.00)

**Table 2:** Distribution of the patients by goniometric measurements of knee ROM (n = 66).

Mobility	Mean ± SD	Range (in degree)
Maximum flexion	126.71 ± 4.88	112.00–135.00
Maximum extension	–3.98 ± 1.74	–2.00 to –10.00
Total excursion	122.71 ± 5.79	104.00–133.00
Extension deficit	3.98 ± 1.74	2.00–10.00
Internal rotation	6.38 ± 1.29	2.00–9.00
External rotation	8.48 ± 1.55	5.00–12.00

**Table 3:** Distribution of the patients by KLs of OA knee (n = 66).

Score	KLs, Medial, n (%)	KLs, Lateral, n (%)	KLs, Patellofemoral, n (%)	Maximal KLs, n (%)
0	0 (0.0)	0 (0.0)	0 (0.0)	0 (0.0)
1	2 (3)	23 (34.8)	18 (27.3)	0 (0.0)
2	23 (34.8)	31 (47.0)	41 (62.1)	25 (37.9)
3	35 (53.0)	12 (18.2)	7 (10.6)	35 (53.0)
4	6 (9.1)	0 (0.0)	0 (0.0)	6 (9.1)
**Total**	**66 (100.0**)	**66 (100.0**)	**66 (100.0**)	**66 (100.0**)
**Mean ± SD**	2.67 ± 0.67	1.84 ± 0.70	1.83 ± 0.58	2.69 ± 0.61

**Table 4:** Pearson correlation coefficients between the knee range of motion values and KLs.

ROM	KLs, medial	KLs, lateral	KLs, patellofemoral	Maximal KLs
Maximum flexion	r = –0.821	r = –0.613	r = –0.539	r = –0.811
	*P* < .001	*P* < .001	*P* < .001	*P* < .001
Maximum extension	r = –0.436	r = –0.430	r = –0.560	r = –0.475
	*P* < .001	*P* < .001	*P* < .001	*P* < .001
Total excursion	r = –0.821	r = –0.644	r = –0.620	r = –0.825
	*P* < .001	*P* < .001	*P* < .001	*P* < .001
Internal rotation	r = –0.469	r = –0.362	r = –0.394	r = –0.469
	*P* < .001	*P* = .003	*P* = .001	*P* < .001
External rotation	r = –0.432	r = –0.481	r = –0.410	r = –0.472
	*P* < .001	*P* < .001	*P`* = 0.001	*P* < .001

**Figure 1.**
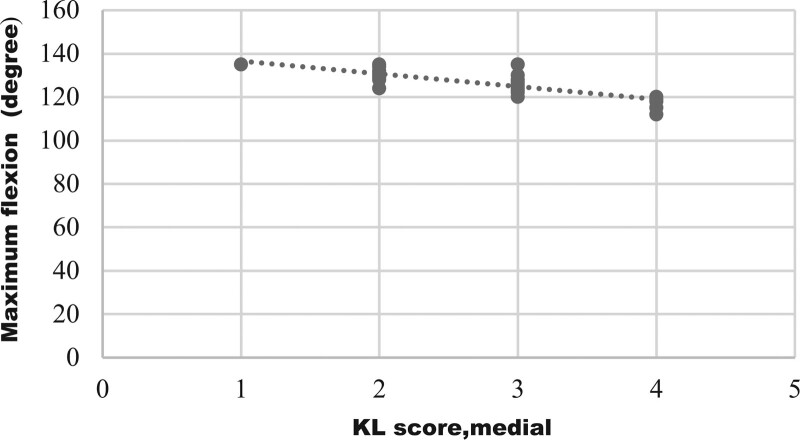
Correlation of maximum flexion with medial compartment KL score. This is the graphical representation of statistically significant negative correlation (strong correlation) between maximum flexion and medial compartment KL score (r = –0.821, *P* ≤ 0.001). It shows with increasing of medial compartment score there was decrease in flexion. KL = Kellgren-Lawrence.

**Figure 2.**
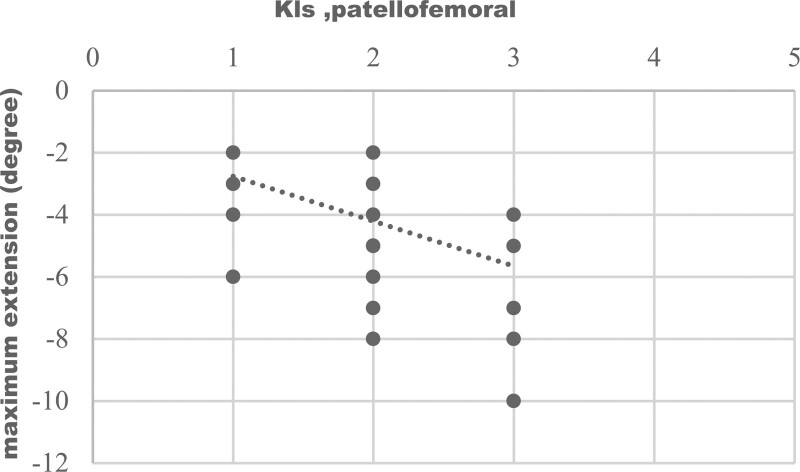
Correlation of maximum extension with KL score patellofemoral. Negative correlation (moderate correlation) was present between maximum extension and the patellofemoral KL score and this relation was statistically significant (r = –0.560, *P* ≤ 0.001). KL = Kellgren-Lawrence.

**Figure 3.**
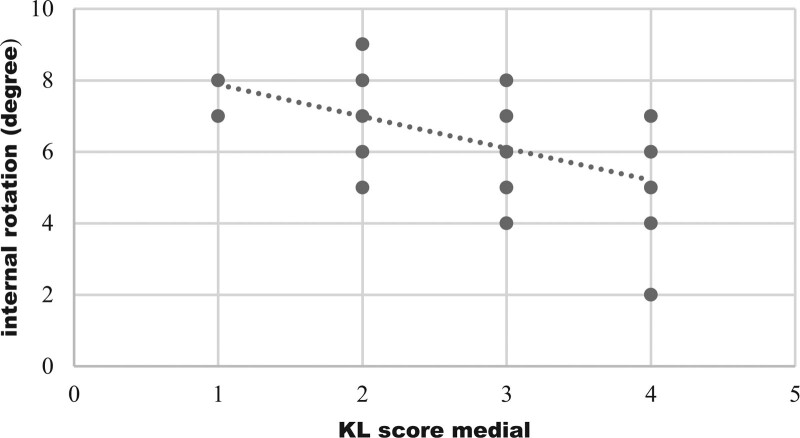
Correlation of internal rotation with medial compartment KL score. Negative correlation (weak correlation) was present between internal rotation and the medial tibiofemoral KL score and this relation was statistically significant (r = –0.469, *P* ≤ 0.001). It shows with advancement of medial compartment scores there was limitation of internal rotation. KL = Kellgren-Lawrence.

**Figure 4.**
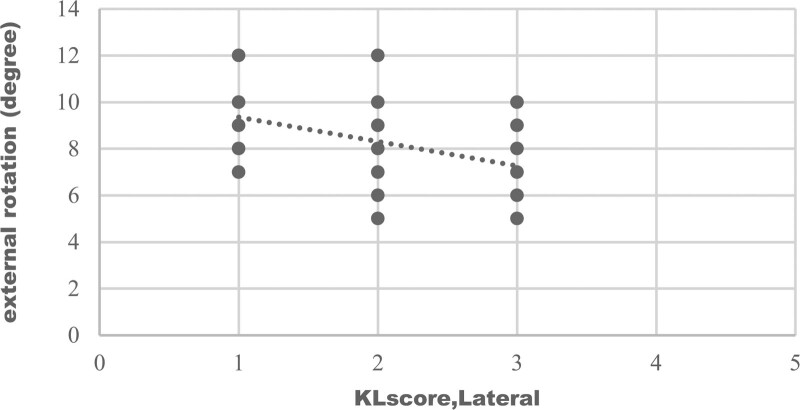
Correlation of external rotation with lateral compartment KL score. There was negative correlation (weak correlation) between external rotation and the lateral tibiofemoral KL score, and this relation was statistically significant (r = –0.481, *P* ≤ 0.001). So with increment of lateral compartment KL score value, there was decreased external rotation. KL = Kellgren-Lawrence.

Graphical representation of correlation (strong correlation) between maximum flexion and medial compartment KL score was shown on Figure 1, which represents, with increasing of medial compartment score there was decrease in flexion. Figure 2 shows negative correlation of maximum extension with patellofemoral compartment scores. With advancement of medial compartment scores there was limitation of internal rotation (Fig. 3). Figure 4 shows with increment of lateral compartment KL score value, there was significant decreased external rotation.

## 4. Discussion

The present cross-sectional study was conducted in a tertiary hospital of Bangladesh (BSMMU) with an aim to measure goniometric ROMs and correlate with KL radiographic score of osteoarthritic knees. For this reason, 66 female primary OA knee patients were selected purposively. Measurements of knee ROM values of the subjects revealed deficit of all ROMs comparing to reference value.^[14]^ As patients with severe pain were excluded; so diminished active ROM was probably due to consequence of disease process but not because of pain. Higher radiographic scores, resulting from articular changes due to disease, were found related with decreased joint motions.

Range of motion of knee joints were measured by a universal goniometer. The mean maximum flexion of the patients was 126.71 **±** 4.88°. Slight variation was observed between study to study. A Turkish study conducted with female OA knee patients reported mean maximum flexion 120.4°,^[15]^ while the study of Massardo et al^[16]^ found the mean maximum flexion of the patients was 128°. Ersoz and Ergun^[10]^ found the mean maximum flexion of the patients was 131.5°. These differences might be due to Ersoz and Ergun included both male and female patients in their study, whereas the patients of the study of Nur et al had higher BMI.^[10,15]^

The mean maximum extension of the patients was –3.98° (**±**1.74), which was consistent with the result of Ersoz and Ergun^[10]^ who showed the mean of maximum extension of the patients was –4° (**±**4.5).

The mean internal rotation was 6.38° (**±**1.29), while external rotation was 8.48° (**±**1.55). These results were in between reference range of classic text books and less than normal limits.^[14]^

Knee joint has 3 compartments. Radiographic score of each joint compartment was determined separately in this study and the highest value among them was determined as maximal score. Greater number of patients had KL radiographic score 3 in the medial compartment, while in lateral compartment, nearly half of the patients had KL radiographic score 2 and in patellofemoral compartment, majority patients had KL radiographic score 2 which matched other study.^[10]^ Massardo et al conducted a study in Bristol, where they found, in OA knee, predominance of medial compartment involvement was more than lateral and patellofemoral compartment, which was evident by radiological changes.^[16]^ According to the study of Ozdemir et al in OA knee, joint space width was lower in medial compartment than lateral compartment.^[17]^ Some other study did weight bearing radiographic view of tibiofibular joints with KL scoring and found majority of subject had score 2 or more, although scoring for each compartment was not done separately.^[15]^

The current study found significant strong to moderate negative correlations between maximum flexion and all KL scores. The study of Ersoz and Ergun also reported significant negative moderate or weak correlation between these parameters. This inconsistency might be due to the small sample size and including both gender in their study. Also, they included doubtful OA (maximal KL score 1) while present study was conducted with all definite OA (maximal KL score 2 or more).

Restriction of extension was related with radiological changes of patellofemoral, medial, and lateral compartments, in this order, resembled by moderate to weak correlation values between maximum extension and KL scores of the compartments. Both rotation movements were negatively correlated with all KL scores. The current study found, limitation of internal rotation was related more with advancement of medial compartment scores, while external rotation restriction was related more with involvement of lateral compartment.

Summation of maximum flexion and maximum extension was determined as total excursion. There were significant negative correlations between total excursion and radiographic score of medial, lateral, and patellofemoral compartment scores.

From another point of view, medial compartment KL score were found negatively correlated with all ROMs and the correlation coefficient value was highest for maximal flexion and then for internal rotation, extension, and external rotation, respectively. During squatting, full knee flexion is required with the tibia maximally internally rotated.^[18]^ In Bangladesh, a large group of population use low commodes. Moniruzzaman et al (2018) found, 73.3% among the respondents use low commode in their study.^[19]^ Symptoms of knee OA typically aggravated by joint use and relieved by rest.^[20]^ Females mostly, do the household chores, kitchen works, offer prayer which usually need squatting and kneeling that may be a reason of medial KLs correlated with maximal flexion and internal rotation mostly. Lateral compartment KLs were related with limitation in flexion, external rotation, extension, and internal rotation, respectively. Ozdemir et al^[17]^ found significant correlation between knee flexion and joint space narrowing in lateral tibiofemoral compartment. Patellofemoral KL scores were correlated mostly with maximum extension, which is similar with the study of Ersoz and Ergun (2003). Maximal KL score was correlated negatively with all knee ROMs, which was consistent with previously mentioned study.

Knee joints bear weight, perform physical activity and exhibit a joint-specific ROM during movement. During OA development, the entire joint organ is affected, including articular cartilage, subchondral bone, synovial tissue, ligaments, menisci, and ultimately leading reduced ROM. Signature pathologic feature of OA is articular cartilage loss, joint disruption, osteophytes formation, and lack of joint space, which can be seen in plain radiograph.^[20,21]^ A reliable radiographic severity grading of OA knee is possible with the application of scoring systems and individual features, especially with well-trained readers.^[22]^ Currently, there are no interventions available to restore degraded cartilage.^[20]^ So earlier diagnosis, both clinically and radiographically will help for further deterioration and also rehabilitation approaches.

In summary of the present study, restricted knee motions were noticed among the respondents. Variable radiographic scores were found in all 3 joint compartments. Medial compartment had comparatively more changes than other 2 compartments. Limitation of flexion and internal rotation can be a clue for predominant medial compartment involvement. On the other hand, damage to patellofemoral compartment resulted in diminished extension more than other movements. A prediction could be done for higher lateral compartment KL scores with decreased flexion and external rotation more than rest of movements.

## 5. Conclusion

There were significant negative correlations between goniometric measurements of knee ROM and radiographic scores in osteoarthritis knee in female patients. So, it can be concluded that KL radiographic scores of separate compartments denotes damage or pathological changes of those area and so there was more limitations of different knee ROMs when the score was more.

## 6. Limitations of study

Some limitations were perceived while performing the study. The following were the limitations of the study:

- The study place was selected purposively which might result in selection bias.
- As the study was conducted in only 1 institution (BSMMU), results might not represent the entire population.
- Cross-sectional design of the study was another limitation.
- Only 66 patients were included in the study, which might not be representing the entire population.
- Only most affected knee was examined, both knee examination and comparison lacking were deficit in this study.

## 7. Strength of the study

This study was an observational study and correlation was done between different variables. All knee ROM were measured by researcher who is a clinician and all x-ray reports were done by single expert radiologist, who was blind about the patients. So, there should be less chance of biasness and interpersonal measurement variations.

All the available knee ROM were measured and correlated with KL scores. Very few studies were available for such relationship assessment. It will help to understand complex relationship between a lot of different variables. There was an endeavor to explore knee OA among the female, as they are more sufferer than male.

## 8. Recommendations

According to the present study findings, the following recommendations were put forward:

- All available ROMs should be measured during clinical examination of OA knee. It may be helpful to assume the compartments affected by OA.
- Radiographic evaluation of each knee joint compartment can suggest about the approximate deficits of ROMs in knee OA. While radiological diagnosis, apart from the maximum KL score, compartment wise scores can also be determined for proper management purpose. Therefore, the Radiology and imaging specialists may be requested to kindly mention all the KL scores.
- As radiographic severity of OA knee increases, ROMs decreases, so earlier comprehensive management and rehabilitation can prevent further ROM and radiological deterioration.
- Large sample size can be taken for more information in future.

## Acknowledgments

Sincere thanks and gratitude to Dr AKM Salek, Professor and Chairman, Dr Moshiur Rahman Khasru, Associate Professor of department of Physical Medicine and Rehabilitation. Grateful to Dr Ahasanul Haque, Dr Shimu Sarkar, Dr Iffat Islam Khan, Dr KM Sayeeduzzaman, Dr Reaz residents of Bangabandhu Sheikh Mujib Medical University (BSMMU), Shahbag, Dhaka for their support. Also, I like to thank DR Mainul Ahsan Associate Professor of Department of Radiology and Imaging, Bangabandhu Sheikh Mujib Medical University (BSMMU), Dhaka for reporting X-ray of knee joint of study population.

## Author contributions

Conceptualization,data curation, investigation, writing original draft, writing review and editing done by Md. Rashid Al Mahmood.

Conceptualisation also done by Professor Taslim uddin, Associate professor Mohammed Tariqul Islam, Shamim MD Fuad.

Editing was helped by Tanvir Rahman shah.

## Supplementary Material


