# Supplementary material for: Correlation between goniometric measurements of range of motion and radiographic scores in osteoarthritis knee: An observational study among females
Source: Medicine (Baltimore). 2022 Aug 12;101(32):e29995. doi: 10.1097/MD.0000000000029995 (PMC9371540; doi:10.1097/MD.0000000000029995)
Supplement: Supplementary file 1 [file medi-101-e29995-s001.pdf]

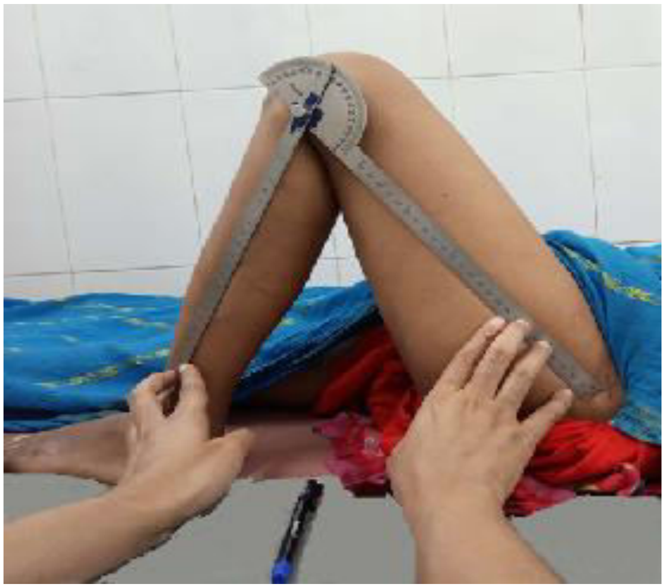

Figure- Measurement of active flexion of knee joint

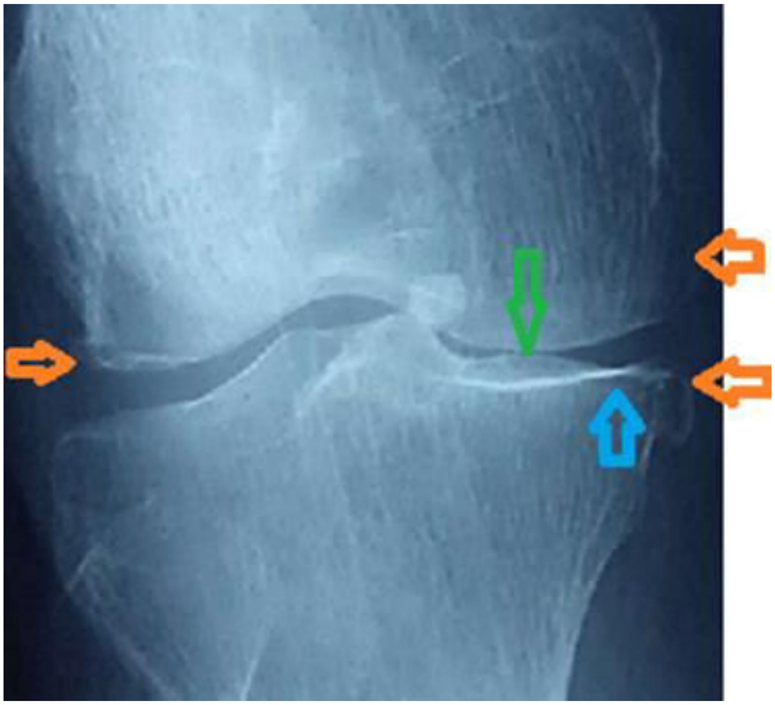

Figure - - KL score -grade 2 in lateral TF and grade 3 in medial TF joint in standing A/P view

Orange arrow -osteophyte, ,blue arrow -marjinal sclerosis, green arrow – joint space narrowing

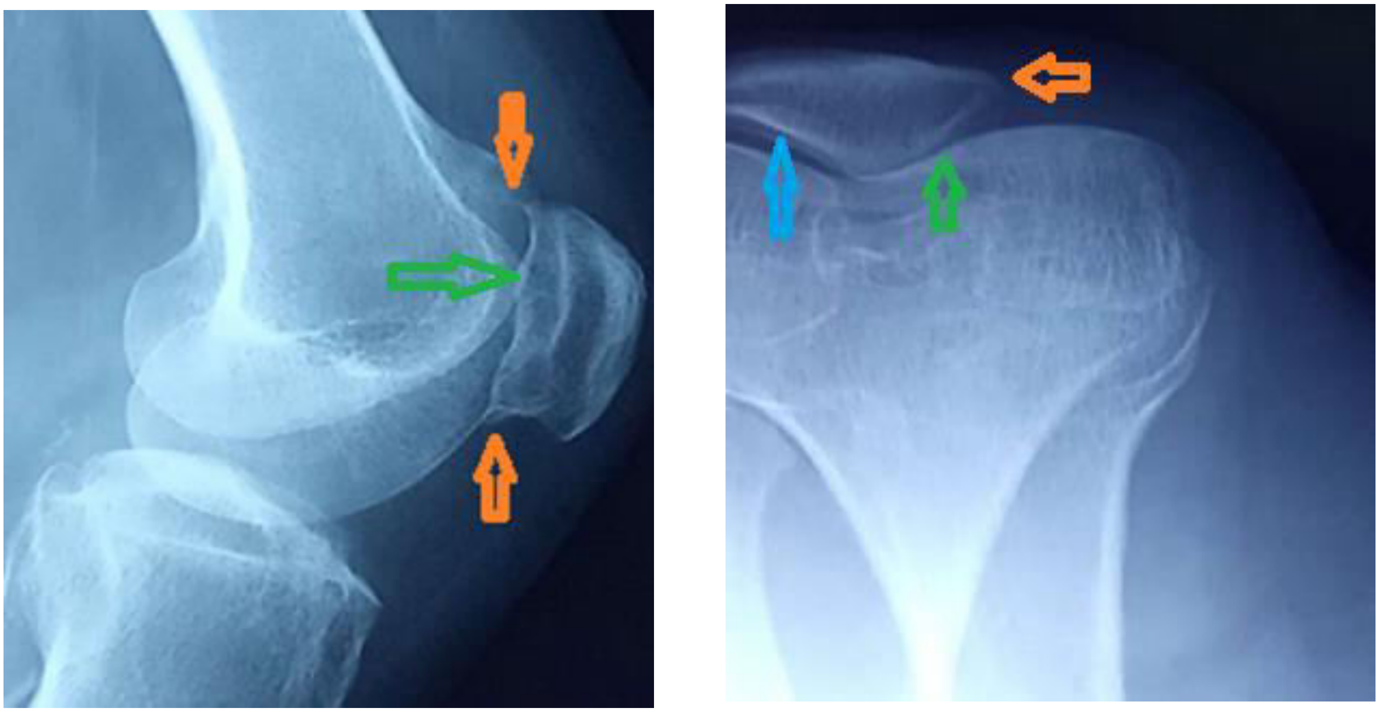

Figure -Patellofemoral joint osteoarthritis in lateral view and skyline view, where KLs score were 2&3 respectively.

ACR criteria for OA knee

American College of Rheumatology Radiologic and Clinical Criteria for Osteoarthritis (ACR):

Clinical:

1. Knee pain for most days of prior month.
2. Crepitus on active joint motion.
3. Morning stiffness of the knee ≤30 minutes.
4. Bony enlargement of the knee on examination
5. Age; ≥38 yrs.

OA knee present when: 1, 2, 4, or 1,2,3,5, or 1, 4, 5 are found.

Clinical & Radiographic:

1. Knee pain for most days of prior month.
2. Radiographic osteophytes at the joint margin.
3. Synovial fluid typical of osteoarthritis. (at least 2: clear, viscous, WBC< 2000 cells/ml).

4. Age ≥40 years.
5. Morning stiffness of the knee ≤30 minutes.
6. Crepitus on active joint motion.

OA knee present when: 1, 2 or 1,3,5,6 or 1,4,5,6 is found.(Nelson and Jordan, 2013)
